# Sensory Flavor Profile of Split Gill Mushroom (*Schizophyllum commune*) Extract and Its Enhancement Effect on Taste Perception in Salt Solution and Seasoned Clear Soup

**DOI:** 10.3390/foods12203745

**Published:** 2023-10-12

**Authors:** Tanwarat Laplamool, Suntaree Suwonsichon, Sarisuk Sittiketgorn, Aussama Soontrunnarudrungsri

**Affiliations:** Kasetsart University Sensory and Consumer Research Center (KUSCR), Department of Product Development, Faculty of Agro-Industry, Kasetsart University, Bangkok 10900, Thailand; tanwarat.lap@ku.th (T.L.); fagisss@ku.ac.th (S.S.); aussama.s@ku.th (A.S.)

**Keywords:** split gill mushroom, taste enhancement, flavor enhancer, sensory perception, salty, umami

## Abstract

Edible mushroom has attracted increasing attention as a natural flavor enhancer. This research studied sensory flavor profiles and identified umami taste-related compounds in split gill mushroom extract (SGME) using descriptive analysis and chemical analysis, respectively. The effects of SGME on taste enhancement as perceived by trained descriptive panelists and general consumers were evaluated in salt solutions and clear chicken soups. The results showed that SGME had mushroom, bitter aromatic, dark brown, meaty, and musty flavor notes and salty and umami tastes. Glutamic acid, aspartic acids, adenosine 5′-monophosphate (5′-AMP), and guanosine 5′-monophosphate (5′-GMP) contributed to SGME’s umami taste. As perceived by trained panelists, saltiness enhancement caused by SGME in aqueous solutions occurred only at relatively low salt concentrations (0.3 and 0.5%), while its umami enhancement effect was more pronounced. When SGME was added into reduced-salt seasoned clear chicken soups, it helped to enhance both the salty and umami tastes of the soups. The 20–31.25% reduced-salt soups with 12.5% of SGME were rated as salty as (*p* > 0.05) the control soup with regular salt content as perceived by both trained panelists and general consumers. The results suggest that SGME could be used as a natural flavor enhancer in the development of reduced-salt foods.

## 1. Introduction

Salt or sodium chloride is one of the most important ingredients used in foods. According to the World Health Organization [1], an average of 10.8 g of salt is consumed per day. The amount is double the recommended maximum intake in adults (5 g of salt per day, which is equivalent to 2 g of sodium per day). Excessive salt consumption increases the risk of non-communicable diseases (NCDs), such as hypertension, cardiovascular diseases, stomach cancer, obesity, osteoporosis, and kidney diseases [1,2]. 

Reducing salt content is a challenging task for the food industry because salt not only contributes to saltiness but also provides texture and microbiological safety of foods [2]. A decrease in salt content often leads to a decrease in consumer acceptance. Several strategies have been used to reduce salt content in foods. The use of flavor enhancers such as monosodium glutamate (MSG) to enhance saltiness perception through its umami taste is one of the promising strategies [3]. However, some consumers consider MSG to be an ‘unhealthy’ and ‘artificial’ food additive [4]. MSG has also been linked to obesity, metabolic disorders, Chinese Restaurant Syndrome, neurotoxic effects, and detrimental effects on reproductive organs [5]. A study by Wang and Adhikari [6] revealed that more than 60% of consumers in the United States tried to avoid or reduce consumption of MSG-containing foods although the FAO and the WHO have declared MSG as safe for consumption. 

Recently, edible mushrooms have attracted great attention as a major source of natural umami components, such as free amino acids and 5′-nucleotides [7]. Studies have proven the success of using varieties of mushrooms as natural flavor enhancers in various food categories, for instance, dried shiitake mushroom stipes (*Lentinula edodes*) in low-salt beef burger [2], shiitake mushroom extract in cooked minced beef [8] and salt-reduced beef burger [9], button mushroom (*Agaricus bisporus*) and oyster mushroom (*Pleurutus ostreatus*) powders in salt- and fat-reduced frankfurter sausage [10], 5′-nucleotide extract of button mushroom in beef soup [11], and winter mushroom (*Flammulina velutipes*) powder in low-salt chicken sausage [12], among others. 

Split gill mushroom (*Schizophyllum commune*) is an edible mushroom that can be found in almost every continent except Antarctica. People in Asian countries such as China, India, Thailand, Taiwan, Malaysia, and Vietnam usually consume split gill mushroom as food and medicine. It is high in protein, dietary fiber, and minerals, such as iron, zinc, and manganese, but low in fat [13]. The mushroom also contains several bioactive compounds that are beneficial to human health, such as phenolic compounds with antioxidant and antidiabetic activities, glucans with antiradical capability, and schizophyllan with antimicrobial, anti-inflammatory, and immune-boosting properties [14]. Additionally, split gill mushroom contains umami amino acids and 5′-nucleotides [15,16]. Based on the authors’ knowledge, only a limited number of studies have investigated the potential use of split gill mushroom as a flavor enhancer in foods, one of which was a study by Hiranpradith et al. [15]. The researchers found that split gill mushroom powder could be used as an alternative ingredient to MSG as it enhanced the umami taste and salinity of reduced-salt soup. However, their findings were based solely on the results of an electronic tongue. Further studies should be conducted to confirm the taste enhancement effect of split gill mushroom as perceived by humans using sensory evaluation. 

The use of split gill mushroom (in the form of an extract) as a natural flavor enhancer was further investigated in the present study. The objectives were (1) to determine the sensory flavor profile and identify umami taste-related compounds of split gill mushroom extract (SGME); (2) to evaluate the effects of SGME on taste enhancement as perceived by highly trained descriptive panelists using a simple system represented by a salt solution and in a complex food system represented by seasoned clear chicken soup; and (3) to evaluate the effects of SGME on taste enhancement as perceived by general consumers in reduced-salt seasoned clear chicken soup.

## 2. Materials and Methods

### 2.1. Materials

#### 2.1.1. Ingredients

Dried split gill mushroom (*Schizophyllum commune*) was purchased from Baanhedkrang farm (Songkhla, Thailand). Salt (iodized refined salt, 99.9% purity, Prung Thip^TM^, Thai Refined Salt Co., Ltd., Nakhon Ratchasima, Thailand), chicken carcass, Chinese radish, coriander root, soy sauce (Recipe 1, Deksomboon^TM^, Yan Wal Yun Corporation Group Co., Ltd., Bangkok, Thailand), white pepper (Raitip^TM^, Thanya Farm Co., Ltd., Nonthaburi, Thailand), and refined sugar (Mitr Phol^TM^, Mitr Phol Sugar Corp., Ltd., Bangkok, Thailand) were purchased from local providers in Thailand. 

#### 2.1.2. Standards and Reagents for Chemical Analysis

Cytidine 5′-monophosphate disodium salt (5′-CMP, AR grade) and uridine 5′-monophosphate disodium salt (5′-UMP, AR grade) were purchased from Alfa Aeser^TM^ (Heysham, UK). Guanosine 5′-monophosphate disodium salt hydrate (5′-GMP, AR grade), methanol (HPLC grade), and ultrapure water were purchased from Fisher Scientific (Loughborough, UK). Inosine 5′-monophosphate disodium salt hydrate (5′-IMP, HPLC grade) and adenosine 5′-monophosphate disodium salt (5′-AMP, HPLC grade) were purchased from Sigma-Aldrich Co., Ltd. (Singapore). Orthophosphoric acid (HPLC grade) was purchased from Loba Chemie^TM^ (Tarapur, India).

### 2.2. Preparation of Split Gill Mushroom Extract (SGME)

The dried split gill mushroom (250 g per batch) was washed with water and drained in a colander. It was then wrapped in a clean cheesecloth and steamed (90–100 °C) for 5 min using a household stainless steel steamer. After steaming, reverse osmosis deionized water (50 mL) was sprayed onto the mushroom. Thereafter, the mushroom was squeezed using a manual fruit press juicer and the obtained extract was used for further study. The amount of mushroom extract obtained from each preparation batch was 200 g. Salt contents of all batches of SGME, as determined using a digital salt meter (PAL-SALT, Atago, Japan) based on the electrical conductivity method, were controlled in a range of 0.60–0.62%. The SGME was kept in a freezer (−18 °C) and thawed before being used for experiments 1–3 within three days after preparation.

### 2.3. Experiment 1

Split gill mushroom extract (SGME) was subjected to descriptive sensory analysis and chemical analysis to determine its flavor profile and umami taste-related compounds, respectively.

#### 2.3.1. Descriptive Sensory Analysis

SGME was evaluated by nine highly trained descriptive panelists to determine its flavor characteristics according to a profile method established by Keane [17], which is the same method used by Pinsuwan et al. [18]. The panelists (all females, age range 39–57 y) were affiliated with Kasetsart University Sensory and Consumer Research Center (KUSCR) and had 120 h of training in descriptive analysis and a minimum of 2200 h of testing experiences with various food products and beverages. The number of panelists fell within the range of 8–12 as suggested by Heymann et al. [19] for descriptive analysis. Sensory evaluations were conducted at the sensory evaluation facilities of KUSCR. The rooms had appropriate lighting, were air conditioned (about 25 °C), and did not have extraneous odors. The testing room was separated from the sample preparation room.

Three 3 h sessions were held to develop a sensory lexicon for evaluating flavor characteristics of SGME. The SGME sample (15 mL) was served in a 2 oz. plastic cup, covered with a lid, and labeled with a three-digit blinding code. Panelists tasted the sample, discussed possible terms, and compiled a final consensus list of attributes for testing. Subsequently, they discussed and reached an agreement on the definition, references, and reference intensities of each attribute. Thereafter, another 3 h session was held during which the panelists individually tasted the mushroom extract and rated the intensity of each attribute on a 15 cm line scale with a score of 0 meaning none, and a score of 15 meaning extremely high. Two separate batches of SGME were evaluated. Reference samples were provided during the evaluations for anchoring intensity values on the scale to minimize panelist-to-panelist variations. Unsalted crackers (Jacob’s Original Cream Cracker, Kraft Foods Malaysia, Petaling Jaya, Malaysia) and reverse osmosis deionized water were provided for panelists to cleanse their palates between samples. The panelists had a 20 min break to minimize the carry-over effect between samples.

#### 2.3.2. Chemical Analysis

The SGME was analyzed for umami taste-related compounds including free amino acids and 5′-nucleotides. Free amino acids were analyzed using an amino acid analyzer at Central Laboratory Co., Ltd. (Bangkok, Thailand) based on two in-house methods, TE-CH-372 [20] and TE-CH-373 [21].

The 5′-nucleotide assay was carried out as described by Hiranpradith et al. [15] using high-performance liquid chromatography (HPLC) (Agilent 6420, Waldbronn, Germany). The mushroom extract was centrifuged at 4000 rpm for 30 min, and then the supernatant was filtered using a 0.22 μm nylon filter prior to HPLC analysis. The 5′-nucleotides were separated on a Kinetex 2.6 μm EVO C18 (100 × 2.10 mm) column for 15 min using an isocratic mobile phase of 5% A and 95% B (A: methanol and B: 0.05% phosphoric acid) at a flow rate of 0.5 mL/min and UV detection at 254 nm. Each 5′-nucleotide was identified by matching its retention time with that of an authentic standard in the HPLC chromatogram and quantified using its respective calibration curve. 

Equivalent umami concentration (EUC), a concentration of monosodium glutamate (MSG) equivalent to the umami intensity given by a mixture of free amino acids and 5′-nucleotides present in the mushroom extract, was calculated using Equation (1) [22]:

(1) $$\;Y=\sum a_{i}b_{i}+1218\;(\sum a_{i}b_{i})\;(\sum a_{j}b_{j})$$

where *Y* is the EUC in terms of g of MSG per 100 g of extract, *a_i_* is the concentration (g/100 g) of each umami amino acid (glutamic acid: Glu and aspartic acid: Asp), *a_j_* is the concentration (g/100 g) of each umami 5′-nucleotide (5′-IMP, 5′-AMP, 5′-GMP, and 5′-XMP), *b_i_* is a constant value for umami amino acids relative to MSG (Glu = 1 and Asp = 0.077), and *b_j_* is a constant value for taste nucleotides relative to that of 5′-IMP (5′-IMP = 1, 5′-AMP = 0.18, 5′-GMP = 2.30, and 5′-XMP = 0.61) [15,23].

#### 2.3.3. Statistical Design and Data Analysis

SGME samples were prepared separately in two batches (duplicate). Sensory evaluation and chemical analysis were performed on each SGME batch. For chemical analysis, two measurements were taken within each batch. Mean values with standard deviations were calculated and presented.

### 2.4. Experiment 2

The effects of SGME on enhancing salty and umami flavors as perceived by highly trained descriptive panelists were evaluated in a simple system represented by salt solution and in a complex food system represented by seasoned clear chicken soup.

#### 2.4.1. Preparation of Test Samples

For a simple solution system, SGME was added into 0.35, 0.5, 0.6, and 0.7% (*w*/*v*) salt solutions at various amounts, namely, 0, 5, 7.5, 10, and 12.5% (*w*/*w*), resulting in a total of 20 salt solution samples for sensory evaluation. 

Ingredients for soup stock preparation were bones from the chicken carcass (450 g), water (3000 mL), sliced Chinese radish, soy sauce (10 g), white pepper powder (5 g), fresh coriander root (4 g), and refined sugar (2 g). All ingredients were added to boiling water in a stainless steel pot; then, the pot was brought to a boil and subsequently simmered for 1 h. The obtained soup stock was refrigerated overnight and, thereafter, chicken fat in the soup stock that became solidified was filtered out. This recipe yielded a soup stock of around 2500 mL (1 batch). Four batches of the soup stock were prepared and combined into a single batch to obtain a uniform soup stock sample for each experimental replication. The stock was reheated and then used to prepare 16 clear soup samples with varying % of added salt (*w*/*w*) and % of SGME (*w*/*w*) as follows:

- Soup sample 1 (control) contained 0.32% of added salt without the addition of SGME.
- Soup samples 2–6 contained 0.26% of added salt (an 18.75% salt reduction compared with the control) with the addition of 0, 5, 7.5, 10, and 12.5% of SGME, respectively.
- Soup samples 7–11 contained 0.24% of added salt (25% salt reduction) with the addition of 0, 5, 7.5, 10, and 12.5% of SGME, respectively.
- Soup samples 12–16 contained 0.22% of added salt (a 31.25% salt reduction) with the addition of 0, 5, 7.5, 10, and 12.5% of SGME, respectively.

The selection of salt content for the control soup was based on preliminary tasting by researchers and the results of salt solutions. 

#### 2.4.2. Descriptive Sensory Analysis

Nine highly trained descriptive panelists (the same group as experiment 1) participated in the test. All sensory evaluations were performed at the sensory evaluation facilities of KUSCR. 

For salt solutions, each sample (15 mL) was served to each panelist at room temperature (25 °C) in a 2 oz. plastic cup that was labeled with a three-digit blinding code. For soups, each sample (20 mL) was served to each panelist in a 4 oz. plastic cup that was labeled with a three-digit blinding code. The serving temperature of soups was controlled in a range of 60–65 °C, following the ASTM standard practice serving protocol for sensory evaluation of soups, sauces, and gravies (E1871-97) [24]. Separate test sessions were conducted for salt solutions and soups. The order of sample serving was randomized across panelists. Panelists individually tasted salt solutions and soups and rated the intensities of salty and umami tastes on 15 cm line scales (0 = none and 15 = extremely high). Reference samples for salty and umami tastes similar to those being used in experiment 1 were provided throughout the evaluations to anchor values on the scales. Unsalted crackers and reverse osmosis deionized water were provided for panelists to cleanse their palates between samples. The panelists had a 10 min break between samples. 

#### 2.4.3. Statistical Design and Data Analysis

The experiment was conducted in two replications and sensory evaluations were completed by replication. Analysis of variance (ANOVA) and Duncan’s multiple range test (DMRT) were performed to compare salty and umami taste intensities among salt solution samples and soup samples. A confidence level of 95% (*p* ≤ 0.05) was adopted in this research. The statistical software being used for all analyses was IBM SPSS Statistics version 28.0 (Thaisoftup Co., Ltd., Bangkok, Thailand).

### 2.5. Experiment 3

The effects of SGME on saltiness enhancement as perceived by general consumers in reduced-salt seasoned clear chicken soup were evaluated.

#### 2.5.1. Preparation of Test Samples

Test samples were selected based on the results of experiment 2 and consisted of three pairs of seasoned clear chicken soups as follows:

- Pair 1: Soup containing 0.32% of added salt without the addition of SGME (control) vs. soup containing 0.26% of added salt (an 18.75% salt reduction compared with the control) with the addition of 12.5% of SGME.
- Pair 2: The control soup vs. soup containing 0.24% of added salt (a 25% salt reduction) with the addition of 12.5% of SGME.
- Pair 3: The control soup vs. soup containing 0.22% of added salt (a 31.25% salt reduction) with the addition of 12.5% of SGME.

The soup samples were prepared and served as described earlier in experiment 2. 

#### 2.5.2. Sensory Evaluation

A total of 64 consumers were invited to participate in the test. To represent the age span, consumers were recruited to form two age groups. The first group consisted of 32 consumers whose ages fell between 18–59 y (18–30 y = 15, 31–45 y = 10, and 46–59 y = 7), while the second group consisted of 32 consumers whose ages fell between 60–80 y (60–70 y = 28 and 71–80 y = 4). 

Three 2-alternative forced choice (2-AFC) tests were performed to determine if a difference in perceived saltiness existed between the two soup samples within each sample pair. For each test, the participants were instructed that they would receive two soup samples, each sample being labeled with a three-digit code. The participants were asked to taste the samples in the order presented from left to right, and then to select the sample they thought was the saltiest. The serving orders of sample pairs and samples within each pair were randomly allocated across the participants. Unsalted crackers and reverse osmosis deionized water were provided for palate cleansing between samples. The participants had a 10 min break between sample pairs. The tests were conducted at the sensory evaluation facilities of KUSCR where each participant sat in an individual booth.

#### 2.5.3. Statistical Design and Data Analysis

Data were analyzed separately for each age group. Within each sample pair, the number of agreeing responses selecting one sample more frequently than another was counted and compared with a critical value in published tables for the 2-AFC test (two-tailed). In this study, the critical value was equal to 23 (*p* ≤ 0.05, *n* = 32) [25]. Therefore, a minimum number of 23 agreeing responses (out of 32 participants) was required to declare a significant difference in perceived saltiness between the two samples within the pair at a 95% confidence level.

## 3. Results and Discussion

### 3.1. Sensory Flavor Profile and Umami Taste-Related Compounds Identified in SGME

The descriptive sensory analysis being used in this research is the most powerful method for capturing product characteristics in terms of perceived attributes and intensities [26]. Details of a sensory lexicon consisting of attributes, definitions, references, and reference intensities developed by the panelists for determining a flavor profile of SGME are shown in Table 1. The developed lexicon facilitated accurate and precise communication regarding SGME’s sensory characteristics across panelists [26].

Seventeen flavor attributes were detected in SGME by the panelists. The intensity scores of all attributes are shown in Table 2. Mushroom identity, bitter aromatic, dark brown, meaty, and musty aromatic notes were perceived at moderate intensity levels (score 5.0–10.0 on a 15-point scale), while briny, sour aromatic, sweet aromatic, earthy, woody, and dry aromatic notes were detected at lower intensity levels (score < 5 on a 15-point scale). For taste, SGME was described as salty and umami. It was also slightly sweet, sour, and bitter. Moreover, the extract caused a slightly astringent sensation.

For umami taste-related compounds, twelve free amino acids were identified in SGME, of which glutamic acid, aspartic acid, arginine, and alanine were more abundant than others (Table 3). Essential amino acids including lysine, leucine, valine, threonine, isoleucine, and histidine were also detected in SGME. The results were consistent with those of Prabsangob and Sittiketgorn [16] and Hiranpradith et al. [15] who identified free amino acid profiles of fresh split gill mushroom and split gill mushroom powder, respectively. Slight differences were observed among the studies for some amino acids. For instance, phenylalanine, methionine, and cysteine were detected in fresh split gill mushroom but not in aqueous extract and dried powder. For 5′-nucleotides, 5′-CMP was most abundant in SGME, followed by 5′-AMP, 5′-GMP, and 5′-UMP, respectively, while 5′-IMP and 5′-XMP were not detected (Table 3). Hiranpradith et al. [15] reported similar types of 5′-nucleotides in split gill mushroom powder. For fresh split gill mushroom, only 5′-CMP, 5′-AMP and 5′-GMP were detected, whereas 5′-UMP was not [16]. The variation in the free amino acid and 5′-nucleotides profiles of fresh split gill mushroom samples could be related to many factors, such as geographic location, cultivation condition, maturity stage, part of mushroom used, and preparation conditions [27].

The presence of MSG-like amino acids including glutamic and aspartic acids and 5′-nucleotides including 5′-AMP and 5′-GMP synergistically contributed to the typical umami taste of SGME [22,27]. The results based on the EUC value indicated that umami intensity per 100 g of SGME was equivalent to the umami intensity of 14.80 g of MSG (Table 3). Mau [28] classified the EUC value into four levels: (1) >1000 g, (2) 100–1000 g, (3) 10–100 g, and (4) <10 g of MSG per 100 g of sample. Therefore, the EUC value of the SGME prepared in the current study was in the third level. The EUC values of split gill mushroom reported in the literature varied in a wide range depending on the forms of mushroom being used for the analysis. For fresh split gill mushroom, the EUC value was 0.1 g of MSG per 100 g (dry weight) sample [16]. The EUC value was much higher for split gill mushroom powder (149.85 g MSG per 100 g of (dry weight) sample) [15]. It should also be mentioned that 5′-CMP, which was detected at the highest concentration of all 5′-nucleotides in split gill mushroom, did not contribute to the EUC quantification based on Equation (1) [22], even though research has shown that the umami taste of mushroom, particularly portobello mushroom, is significantly related to 5′-CMP [29]. 

Apart from the umami taste, the presence of 5′-nucleotides also contributes to the meaty flavor of SGME [30]. Flavor notes such as mushroom identity, earthy, musty, and woody could possibly be related to 1-octene-3-ol, the volatile compound that is naturally present in mushroom [2]. The perception of bitterness and astringency could be attributed to free amino acids with surface hydrophobicity, such as arginine, valine, and isoleucine [30] as well as tannin [31], which were present in split gill mushroom. According to Prabsangob and Sittiketgorn [16], the sweetness of SGME could be linked to trehalose, glucose, and mannitol, while its sourness could be related to succinic, malic, citric, and fumaric acids.

### 3.2. Effects of SGME on Enhancing Salty and Umami Tastes as Perceived by Trained Panelists

#### 3.2.1. Salt Solutions

The effects of SGME on enhancing the perception of salty and umami tastes were evaluated by trained descriptive panelists using 0.35–0.7% (*w*/*v*) salt solutions with 0–12.5% (*w*/*w*) added SGME. The concentrations of salt in solutions being used in this research covered the intensity range of saltiness expected to present in various food categories and covered the whole standard salty scale based on the Spectrum^TM^ (Sensory Spectrum, Inc., New Providence, NJ, USA) descriptive analysis method [25]. Table 4 shows the mean intensities of salty and umami tastes of the salt solutions.

As per Table 4, 0.35% salt solutions with 7.5, 10, and 12.5% of SGME were rated significantly higher (*p* ≤ 0.05) in saltiness than those without SGME. Increasing the amount of SGME from 7.5 to 12.5% tended to increase the saltiness of the 0.35% salt solutions although the difference did not reach significance (*p* > 0.05). For the 0.5% salt solutions, a significant increase (*p* ≤ 0.05) in saltiness was observed only when SGME was added at a 12.5% level. While for the 0.6 and 0.7% salt solutions, the addition of SGME at all levels did not enhance saltiness. When comparing the 0.35 and 0.5% salt solutions, both with 12.5% of SGME, it was found that the degree of increase in salty scores caused by SGME was higher (*p* ≤ 0.05) for the former (Δ = 6.16 − 4.67 = 1.49) than for the latter (Δ = 9.06 − 8.25 = 0.81). Thus, it can be concluded that saltiness enhancement caused by SGME in aqueous solutions occurred only at relatively low concentrations of salt (e.g., 0.3 and 0.5% salt) and that the lower the salt concentration, the higher the effectiveness of SGME. Previous studies that investigated the effects of different odors on saltiness enhancement also reported consistent findings showing that the enhancement occurred only at relatively low salt concentrations (e.g., 0–0.16 M, 0.3%), but not at higher salt concentrations (e.g., 0.04–0.64 M, 0.8%), depending on the studies [32,33].

Unlike saltiness, the addition of SGME at all levels significantly increased the umami intensities of all salt solutions (Table 4). For the 0.35 and 0.5% salt solutions, umami ratings increased (*p* ≤ 0.05) with each increase in SGME level. For the 0.6 and 0.7% salt solutions, the maximum umami ratings were achieved with the addition of 10% of SGME. Increasing the amount of SGME from 10 to 12.5% did not further increase the umami intensities of those solutions. It should also be noted that the umami intensities of salt solutions with SGME were generally higher than that of the SGME solution alone. For instance, the umami rating of the 0.35% salt solution with 12.5% of SGME was 3.44, while that of the 12.5% SGME solution alone with no salt was only 2.80.

The results suggest that salt synergistically enhanced the umami perception of SGME. While SGME enhanced the saltiness of salt solutions simply because the SGME itself was salty, no synergistic enhancement effect of the umami compounds present in SGME on the saltiness of salt solutions was observed in this study.

#### 3.2.2. Seasoned Clear Chicken Soups

The effects of SGME on enhancing the perception of salty and umami tastes were further evaluated in a more complex system, namely, seasoned clear chicken soups containing regular salt content (0.32% of salt or a 0% salt reduction, control) and reduced-salt contents (0.26% of salt or an 18.75% salt reduction; 0.24% of salt or a 25% salt reduction; and 0.22% of salt or a 31.25% salt reduction). SGME was added to these soup samples at 0–12.5% levels. The selection of salt content at the 0.32% level for the control soup was based on preliminary tasting by researchers and the results of salt solution evaluations (Section 3.2.1) showing that SGME enhanced saltiness perception more effectively in the 0.3% salt solution than in solutions with higher salt concentrations. The mean intensities of salty and umami tastes of the soup samples as determined by a trained descriptive panel are shown in Table 5. 

As expected, all reduced-salt soups with no SGME added were rated less salty (*p* ≤ 0.05) than the control soup with regular salt content (Table 5). Saltiness ratings of reduced-salt soups significantly increased (*p* ≤ 0.05) with the addition of 5–12.5% of SGME, when compared with the same salt levels. Such enhancement effect generally increased with increased SGME levels regardless of salt levels in the soup samples. It should also be noted that all reduced-salt soups (18.75–31.25% salt reduction) with 12.5% of SGME were rated as salty as (*p* > 0.05) the control soup with regular salt content. 

Umami taste was detected at low-intensity levels in all soups with no SGME added (Table 5); the taste was possibly derived from other ingredients that were used for the preparation of soups such as chicken bone, Chinese radish, and soy sauce. The addition of SGME further increased umami ratings of all reduced-salt soups. This enhancement effect tended to increase with increased SGME levels.

The results of salty and umami enhancement effects of SGME observed in all reduced-salt soup samples (Table 5) whose salt levels fell in the range of 0.22–0.26% were aligned with those observed in the 0.3% salt solution (Table 4). 

### 3.3. Effects of SGME on Saltiness Enhancement as Perceived by General Consumers in Soup

The effects of SGME on saltiness enhancement as perceived by general consumers in reduced-salt seasoned clear chicken soup were investigated using 2-AFC tests. Three pairs of soup samples were evaluated. Each pair consisted of the control soup with regular salt content and 18.75, 25, or 31.25% reduced-salt soups with the addition of SGME at a 12.5% level. The results showed that the saltiness of the 18.75, 25, or 31.25% reduced-salt soup with SGME was not significantly different (*p* > 0.05) from that of the control soup with regular salt content as perceived by consumers in both age groups (Table 6). The results were in accordance with those of trained panelists who found no significant differences (*p* > 0.5) in saltiness ratings of the soup samples within pairs (Table 5). Therefore, the taste enhancement effect of SGME could be used to compensate for sodium reduction in foods.

## 4. Conclusions

This research studied sensory flavor profiles and identified umami taste-related compounds of SGME. The effects of SGME on taste (salty and umami) enhancement as perceived by highly trained descriptive panelists and general consumers were evaluated in a simple system represented by salt solution and in a complex food system represented by seasoned clear chicken soup. The results show that the flavor of SGME could be described as having mushroom, bitter aromatic, dark brown, meaty, and musty notes as well as salty and umami tastes. Glutamic acid, aspartic acids, and 5′-nucleotides including 5′-AMP and 5′-GMP contributed to the typical umami taste of SGME. As perceived by trained panelists, saltiness enhancement caused by SGME in aqueous solutions occurred only at relatively low concentrations of salt (e.g., 0.3 and 0.5% salt), while its umami enhancement effect was more pronounced. When SGME was used as a flavor enhancer in reduced-salt seasoned clear chicken soup, it helped to enhance both the salty and umami tastes of the soups. The 18.75–31.25% reduced-salt soups with 12.5% of SGME were rated as salty as (*p* > 0.05) the control soup with regular salt content as perceived by both trained panelists and general consumers. The results based on experiments 1–3 suggest that SGME could be used as a natural flavor enhancer in the development of reduced-salt foods. An acceptance test should be further conducted to determine consumer liking of reduced-salt soups with SGME.

## Figures and Tables

**Table 1:** Attributes, definitions, and references for flavor evaluation of split gill mushroom extract.

Attribute	Definition	Reference	Reference Preparation	Reference Intensity
Aromatics				
Mushroom identity	Flavor characteristic of mushrooms consists of earthy, damp, and musty notes with a slightly sour note	Dry shiitake mushroom (Tontawan brand)	Soak 20 g of sliced shiitake mushroom in 200 mL of hot water for 15 min and then finely blend the mixture to obtain a mushroom paste	8.5
Briny	Flavor associated with saltiness and moistness such as ocean water and ocean air	Seasoning soy sauce (Maggi brand)	Mix 0.4 g of seasoning soy sauce in 200 mL of water Mix 1.0 g of seasoning soy sauce in 200 mL of water	2.0 4.0
Bitter aromatic	Flavor associated with the impression of bitterness	Espresso roast coffee powder (Nescafé Red Cup)	Mix 0.167 g of coffee powder in 200 mL of water Mix 1 g of coffee powder in 200 mL of water	5.5 7.0
Sour aromatic	Sharp and pungent flavor that suggests a product would taste sour	Tomato paste (Mica brand)	Mix 6 g of tomato paste in 300 mL of water Mix 24 g of tomato paste in 300 mL of water	2.5 6.5
Sweet aromatic	Flavor associated with the impression of all sweet substances such as fruit, flowers, molasses, candy, caramelized sugar, and maple syrup.	Brown sugar (Mitr Phol brand)	Mix 20 g of brown sugar in 200 mL of water	3.5
Dark brown	Flavor associated with food that has been heated until it almost burns	Chocolate syrup (Hershey’s brand)	Mix 5 g of chocolate syrup in 200 mL of water Chocolate syrup (Pure)	5.5 9.0
Meaty	Flavor associated with meats or meat proteins consisting of briny, savory, metallic, sweet aromatic, and brown notes	Essence of chicken—original formulation (Brand’s brand)	Mix 50 mL of essence of chicken in 200 mL of water Essence of chicken (pure)	4.0 8.0
Earthy	Flavor associated with damp soil, beetroot, or slightly undercooked boiled potato	Beetroot	Mix 40 g of chopped beetroot (2 × 2 mm) in 200 mL water, filter, and only use the filtrate Chopped beetroot (2 × 2 mm)	4.0 7.0
Musty	Flavor associated with closed air spaces or poorly ventilated areas	High-fiber wheat bran cereal (Kellogg’s brand)	Wheat bran cereal (3 pieces)	3.5
Woody	Flavor associated with the bark of a tree	Premium shelled walnut (Heritage brand)	Walnut (1 piece)	4.0
Dry	A dry flavor of a food product as a result of the drying or dehydration process	Soya milk powder (Doi Kham brand)	Mix 1 g of soya milk powder in 200 mL of water Soya milk powder (pure)	3.0 7.0
Tastes				
Sweet	A fundamental taste sensation of which sucrose is typical	Sucrose solution	20 g/L sucrose solution 50 g/L sucrose solution	2.0 5.0
Sour	A fundamental taste sensation of which citric acid is typical	Citric acid solution	0.15 g/L citric acid solution	1.5
Salty	A fundamental taste sensation of which sodium chloride is typical	Sodium chloride solution	2 g/L sodium chloride solution 3.5 g/L sodium chloride solution 5.0 g/L sodium chloride solution 7.0 g/L sodium chloride solution	2.5 5.0 8.5 15.0
Bitter	A fundamental taste sensation of which caffeine is typical	Caffeine solution	0.5 g/L caffeine solution	2.0
Umami	A sweet and salty taste that naturally occurs in some mushrooms, seaweed, and meats	Monosodium glutamate solution	2 g/L monosodium glutamate solution 3.5 g/L monosodium glutamate solution	2.5 5.0
Chemical feeling factor				
Astringent	The complex of drying, puckering, shrinking sensations in the oral cavity	Alum solution	0. 35 g/L alum solution	1.5

**Table 2:** Intensity scores (means ± standard deviations) of flavor attributes detected in the split gill mushroom extract.

Aromatics		Aromatics	
Mushroom identity	7.48 ± 0.80	Meaty	6.65 ± 0.72
Briny	4.85 ± 0.83	Earthy	2.97 ± 0.75
Bitter aromatic	5.85 ± 0.73	Musty	5.69 ± 0.72
Sour aromatic	1.91 ± 0.64	Woody	2.67 ± 0.73
Sweet aromatic	1.38 ± 0.48	Dry	2.72 ± 0.68
Dark brown	7.30 ± 0.49		
**Tastes**		**Chemical feeling factor**	
Sweet	1.62 ± 0.59	Astringent	1.54 ± 0.34
Sour	1.18 ± 0.35		
Salty	7.26 ± 0.82		
Bitter	2.83 ± 0.78		
Umami	4.14 ± 0.49		

**Table 3:** Free amino acids and 5′-nucleotide contents (mean + standard deviation) of split gill mushroom extract.

Amino Acids (mg/100 g Extract)			
Glutamic acid	426.47 ± 8.30	Valine	129.88 ± 2.03
Aspartic acid	198.56 ± 1.41	Glycine	123.20 ± 1.62
Arginine	182.16 ± 0.66	Threonine	107.86 ± 1.69
Alanine	167.99 ± 3.52	Serine	106.26 ± 1.46
Lysine	133.10 ± 0.11	Isoleucine	80.28 ± 1.04
Leucine	129.99 ± 2.26	Histidine	60.17 ± 0.25
**5′-nucleotides (mg/100 g extract)**			
5′-CMP	121.30 ± 0.65		
5′-AMP	29.78 ± 1.51		
5′-GMP	8.39 ± 1.34		
5′-UMP	4.32 ± 1.83		
**Equivalent umami concentration (EUC) (g MSG/100 g extract)**			14.80 ± 2.83

**Table 4:** Salty and umami taste intensities of salt solutions at various concentrations as affected by the addition of split gill mushroom extract.

Tastes	Concentration of Salt Solutions (% *w*/*v*)	Added Split Gill Mushroom Extract (% *w*/*w*)				
		0	5	7.5	10	12.5
Salty	0	0.00 ± 0.00 ^p^	1.34 ± 0.33 ^o^	1.83 ± 0.56 ^n^	2.13 ± 0.69 ^lm^	2.11 ± 0.50 ^l^
	0.35	4.67 ± 0.45 ^j^	3.81 ± 0.53 ^k^	5.92 ± 0.49 ^i^	5.82 ± 0.78 ^i^	6.16 ± 0.65 ^i^
	0.5	8.25 ± 0.26 ^g^	7.72 ± 0.60 ^h^	7.67 ± 0.82 ^h^	8.42 ± 0.69 ^g^	9.06 ± 0.78 ^f^
	0.6	11.78 ± 0.60 ^c^	9.25 ± 0.88 ^f^	9.89 ± 0.70 ^e^	9.36 ± 0.85 ^f^	9.86 ± 0.89 ^e^
	0.7	14.53 ± 0.36 ^a^	12.39 ± 0.87 ^b^	10.72 ± 0.62 ^d^	10.69 ± 0.60 ^d^	10.03 ± 0.80 ^e^
Umami	0	0.00 ± 0.00 ^j^	1.57 ± 0.30 ^i^	2.00 ± 0.37 ^h^	2.72 ± 0.60 ^def^	2.80 ± 0.46 ^cdef^
	0.35	0.00 ± 0.00 ^j^	2.35 ± 0.45 ^fg^	2.00 ± 0.75 ^h^	3.04 ± 0.57 ^bcd^	3.44 ± 0.45 ^a^
	0.5	0.00 ± 0.00 ^j^	1.86 ± 0.43 ^h^	2.51 ± 0.49 ^ef^	2.76 ± 0.55 ^de^	3.08 ± 0.73 ^bc^
	0.6	0.00 ± 0.00 ^j^	2.06 ± 0.80 ^gh^	2.57 ± 0.68 ^def^	3.09 ± 0.62 ^bc^	3.08 ± 0.73 ^bc^
	0.7	0.00 ± 0.00 ^j^	2.07 ± 0.76 ^gh^	2.87 ± 0.65 ^bcde^	3.13 ± 0.69 ^b^	3.04 ± 0.48 ^bcd^

^a–p^ Salty intensities with different letters are significantly different based on ANOVA and DMRT (*p* ≤ 0.05). ^a–j^ Umami intensities with different letters are significantly different based on ANOVA and DMRT (*p* ≤ 0.05).

**Table 5:** Salty and umami taste intensities of seasoned clear soups at various salt reduction levels as affected by the addition of split gill mushroom extract.

Tastes	% Salt Reduction	Added Split Gill Mushroom Extract (% *w*/*w*)				
		0	5	7.5	10	12.5
Salty	0	5.00 ± 0.57 ^ab^	-	-	-	-
	18.75	4.11 ± 0.75 ^g^	4.89 ± 0.71 ^bcd^	4.67 ± 0.78 ^cde^	4.92 ± 0.74 ^bcd^	5.27 ± 0.85 ^a^
	25	3.78 ± 0.24 ^h^	4.40 ± 0.74 ^ef^	4.64 ± 0.69 ^de^	4.82 ± 0.64 ^bcd^	5.08 ± 0.60 ^ab^
	31.25	3.65 ± 0.64 ^h^	4.19 ± 0.68 ^fg^	4.49 ± 0.71 ^e^	4.96 ± 0.86 ^abc^	5.00 ± 0.77 ^ab^
Umami	0	1.51 ± 0.43 ^h^	-	-	-	-
	18.75	1.46 ± 0.35 ^h^	2.44 ± 0.34 ^def^	2.51 ± 0.52 ^cde^	2.57 ± 0.65 ^bcde^	2.71 ± 0.57 ^abc^
	25	0.84 ± 0.29 ^i^	1.86 ± 0.31 ^g^	2.40 ± 0.73 ^ef^	2.46 ± 0.79 ^def^	2.78 ± 0.88 ^ab^
	31.25	1.31 ± 0.40 ^h^	2.22 ± 0.55 ^f^	2.46 ± 0.35 ^def^	2.67 ± 0.54 ^abcd^	2.87 ± 0.60 ^a^

^a–i^ Salty intensities with different letters are significantly different based on ANOVA and DMRT (*p* ≤ 0.05). ^a–h^ Umami intensities with different letters are significantly different based on ANOVA and DMRT (*p* ≤ 0.05).

**Table 6:** Effects of split gill mushroom extract (SGME) on enhancing salty taste in reduced-salt soups as perceived by general consumers based on 2-alternative forced choice (2-AFC) tests.

Pair	Soup Samples within Pair	Number of Consumers Who Selected Each Soup Sample as a Saltier Sample #	
		Young Consumers (18–59 y) (*n* = 32)	Elderly Consumers (>60 y) (*n* = 32)
1	0% salt reduction	11 ^a^	12 ^a^
	18.75% salt reduction with 12.5% of SGME	21 ^a^	20 ^a^
2	0% salt reduction	14 ^a^	16 ^a^
	25% salt reduction with 12.5% of SGME	18 ^a^	16 ^a^
3	0% salt reduction	14 ^a^	14 ^a^
	31.25% salt reduction with 12.5% of SGME	18 ^a^	18 ^a^

# A minimum number of 23 agreeing responses (out of 32 participants) was required to declare a significant difference between the two samples within a pair (*p* ≤ 0.05). ^a^ The number of consumers within the age group who selected one sample over another within the pair was not significantly different (*p* > 0.05).

## Data Availability

The data in this study are available in the article.

## References

[1] Massive Efforts Needed to Reduce Salt Intake and Protect Lives. https://www.who.int/news/item/09-03-2023-massive-efforts-needed-to-reduce-salt-intake-and-protect-lives.

[2] França F., Harada-Padermo S.S., Frasceto R.A., Saldaña E., Lorenzo J.M., Vieira T.M.F.S., Selani M.M. (2022). Umami ingredient from shiitake (*Lentinula edodes*) by-products as a flavor enhancer in low-salt beef burgers: Effects on physicochemical and technological properties. LWT Food Sci. Technol..

[3] Maluly H.D.B., Arisseto-Bragotto A.P., Reyes F.G.R. (2017). Monosodium glutamate as a tool to reduce sodium in food stuffs: Technological and safety aspects. Food Sci. Nutr..

[4] Selani M.M., Ramos P.H.B., Patinho I., França F., Harada-Padermo S.d.S., Contreras-Castillo C.J., Sadaña E. (2022). Consumer’s perception and expected liking of labels of burgers with sodium reduction and addition of mushroom flavor enhancer. Meat Sci..

[5] Niaz K., Zaplatic E., Spoor J. (2018). Extensive use of monosodium glutamate: A threat to public health?. EXCLI J..

[6] Wang S., Adhikari K. (2018). Consumer perceptions and other influencing factors about monosodium glutamate in the United States. J. Sens. Stud..

[7] Sun L.B., Zhang Z., Xin G., Sun B., Bao X., Wei Y., Zhao X., Xu H. (2020). Advances in umami taste and aroma of edible mushroom. Trends Food Sci. Technol..

[8] Dermiki M., Phanphensophon N., Mottram D.S., Methven L. (2013). Contributions of non-volatile and volatile compounds to the umami taste and overall flavour of shiitake mushroom extracts and their application as flavour enhancers in cooked minced meat. Food Chem..

[9] Mattar T.V., Gonçalves C.S., Pereira R.C., Faria M.A., de Souza V.R., Carneiro J.D.D.S. (2018). A shiitake mushroom extract as a viable alternative to NaCl for a reduction in sodium in beef burgers: A sensory perspective. Br. Food J..

[10] Ceŕon-Guevara M.I., Rangel-Vargas E., Lorenzo J.M., Bermúdez R., Pateiro M., Rodríguez J.A., Sánchez-Ortega I., Santos E.M. (2020). Reduction of salt and fat in frankfurter sausages by addition of *Agaricus bisporus* and *Pleurotus ostreatus* flour. Foods.

[11] El-Aleem F.S.A., Taher M.S., Lotfy S.N., El-Massry K.F., Fadel H.H.M. (2017). Influence of extracted 5-nucleotides on aroma compounds and flavour acceptability of real beef soup. Int. J. Food Prop..

[12] Jo K., Lee J., Jung S. (2018). Quality characteristics of low-salt chicken sausage supplemented with a winter mushroom powder. Korean J. Food Sci. Anim. Resour..

[13] Singh S., Raj C., Singh H.K., Avasthe R.K., Said P., Balusamy A., Sharma S.K., Lepcha S.C., Kerketta V. (2021). Characterization and development of cultivation technology of wild split gill *Schizophyllum commune* mushroom in India. Sci. Hortic..

[14] Klaus A., Kozarski M., Niksic M., Jakovljevic D., Todorovic N., Van Griensven D. (2011). Antioxidant activities and chemical characterization of polysaccharides extracted from the Basidiomycete Schizophyllum commune. LWT Food Sci. Technol..

[15] Hiranpradith V., Therdthai N., Soontrunnarudrungsri A. (2023). Effect of steaming and microwave heating on taste of clear soup with split-gill mushroom powder. Foods.

[16] Prabsangob N., Sittiketgorn S. (2023). Profile of taste-related compounds and bioactivity of split gill mushroom (*Schizophyllum commune*) as affected by blanching and drying. Int. J. Food Prop..

[17] Keane P., Hootman R.C. (1992). The flavor profile. Manual on Descriptive Analysis Testing for Sensory Evaluation.

[18] Pinsuwan A., Suwonsichon S., Chompreeda P., Prinyawiwatkul W. (2022). Sensory drivers of consumer acceptance, purchase intent and emotions toward brewed black coffee. Foods.

[19] Heymann H., Machado B., Torri L., Robinson A.L. (2012). How many judges should one use for sensory descriptive analysis?. J. Sens. Stud..

[20] Official Journal of the European Communities (1983). L 257.

[21] Çevikkalp S.A., Löker G.B., Yaman M., Amoutzopoulos B. (2016). A simplified HPLC method for determination of tryptophan in some cereals and legumes. Food Chem..

[22] Yamaguchi S., Yoshikawa T., Ikeda S., Ninomiya T. (1971). Measurement of the relative taste intensity of some α-amino acids and 5′-necleotides. J. Food Sci..

[23] Cho I.H., Choi H., Kim Y. (2010). Comparison of umami-taste active components in pileus and stipe of pine-mushrooms (*Tricholoma matsutake* Sing.) of different grades. Food Chem..

[24] (1997). Standard Practice for Serving Protocol for Sensory Evaluation of Foods and Beverages.

[25] Meilgaard M., Civille G.V., Carr B.T. (2007). Sensory Evaluation Techniques.

[26] Suwonsichon S. (2019). The importance of sensory lexicons for research and development of food products: A Review. Foods.

[27] Zhang Y., Venkitasamy C., Pan Z., Wang W. (2013). Recent developments on umami ingredients of edible mushrooms—A review. Trends Food Sci. Technol..

[28] Mau J.L. (2005). The umami taste of edible and medicinal mushrooms. Int. J. Med. Mushrooms.

[29] Wang S., Tonnis B.D., Wang M.L., Zhang S., Adhikari K. (2019). Investigation of monosodium glutamate alternatives for content of umami substances and their enhancement effects in chicken soup compared to monosodium glutamate. J. Food Sci..

[30] Zhao C.J., Schieber A., Gänzle M.G. (2016). Formation of taste-active amino acids, amino acid derivatives and peptides in food fermentation—A review. Food Res. Int..

[31] Yusran Y., Erniwati E., Khumaidi A., Pitopang R., Radix I., Jati A.P. (2023). Diversity of substrate type, ethnomycology, mineral composition, proximate, and phytochemical compounds of the *Schizopyllum commune* Fr. in the area along Palu-Koro Fault, Central Sulawesi, Indonesia. Saudi J. Biol. Sci..

[32] Linscott T.D., Lim J. (2016). Retronasal odor enhancement by salty and umami tastes. Food Qual. Prefer..

[33] Zhou T., Fenga Y., Danguinb T.T., Zhao M. (2021). Enhancement of saltiness perception by odorants selected from Chinese soy sauce: A gas chromatography/olfactometry-associated taste study. Food Chem..

